# Supplementary figures and images for: Virtual reality vs. Tablet video for venipuncture education in children: A randomized clinical trial
Source: PLoS One. 2024 Aug 27;19(8):e0307488. doi: 10.1371/journal.pone.0307488 (PMC11349209; doi:10.1371/journal.pone.0307488)

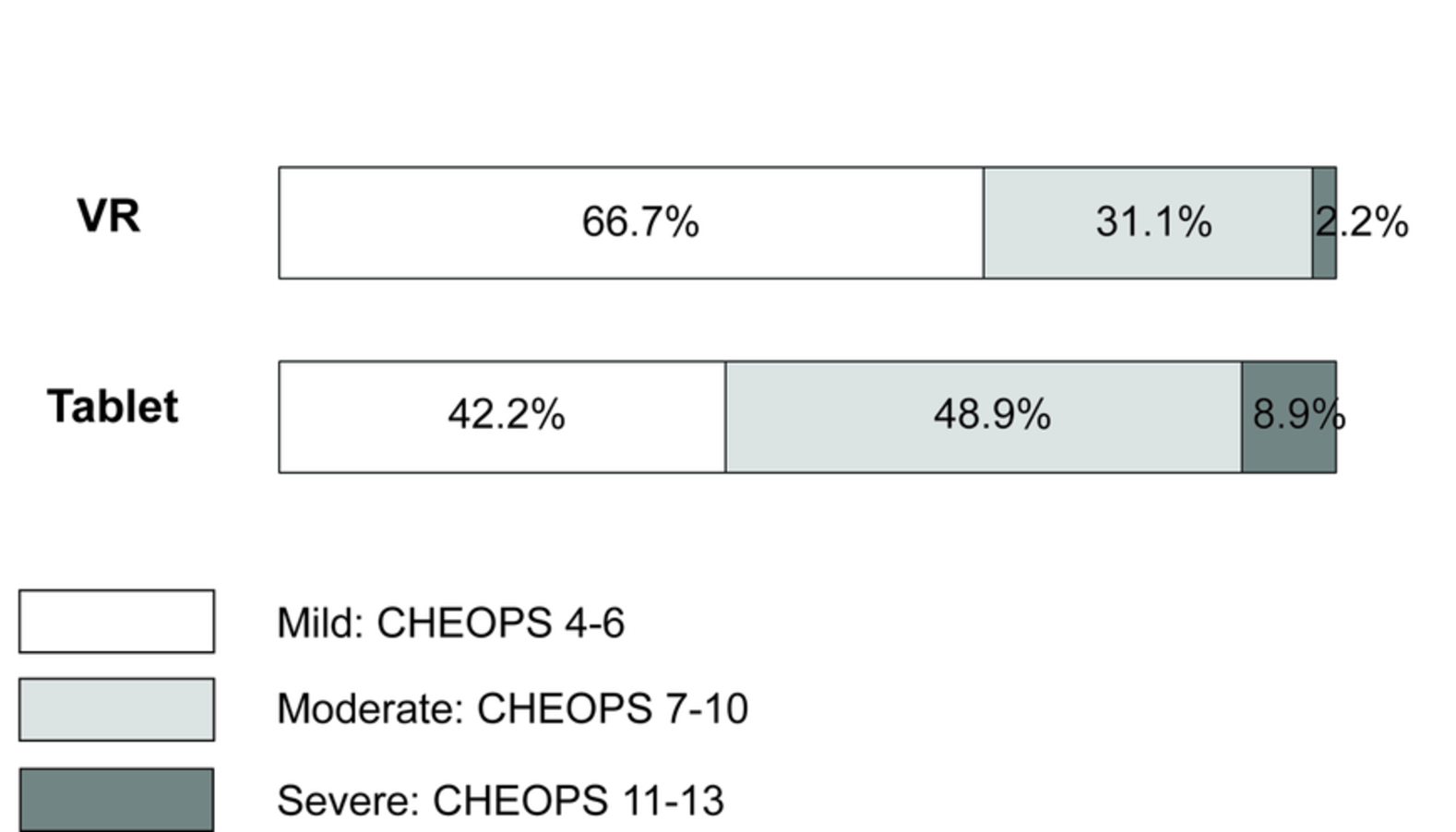

Supplement: S1 Fig — CHEOPS, Children’s Hospital of Eastern Ontario Pain Scale. (TIF) [file pone.0307488.s001.tif]
