# Supplementary material for: Virtual reality vs. Tablet video for venipuncture education in children: A randomized clinical trial
Source: PLoS One. 2024 Aug 27;19(8):e0307488. doi: 10.1371/journal.pone.0307488 (PMC11349209; doi:10.1371/journal.pone.0307488)
Supplement: S1 Table — (DOCX) [file pone.0307488.s002.docx]

**Supporting table 1** Children’s hospital of eastern ontario pain scale (CHEOPS)

| **Criteria** | | **Score** |
| --- | --- | --- |
| **Cry** | No cry | 1 |
|  | Moaning/Crying | 2 |
|  | Screaming | 3 |
| **Facial expression** | Smiling | 0 |
|  | Composed | 1 |
|  | Grimace | 2 |
| **Verbal** | Positive | 0 |
|  | None/Other complaints | 1 |
|  | Pain complaints | 2 |
| **Torso** | Neutral | 1 |
|  | Shifting/Tense/Shivering/Upright/Restrained | 2 |
| **Touch** | Not touching | 1 |
|  | Reach/Touch/Grab/Restrained | 2 |
| **Legs** | Neutral | 1 |
|  | Squirming/Kicking/Drawn Up/Tensed/Standing/Restrained | 2 |
