# Supplementary material for: Virtual reality vs. Tablet video for venipuncture education in children: A randomized clinical trial
Source: PLoS One. 2024 Aug 27;19(8):e0307488. doi: 10.1371/journal.pone.0307488 (PMC11349209; doi:10.1371/journal.pone.0307488)
Supplement: S1 Protocol — (DOCX) [file pone.0307488.s004.docx]

**STUDY PROTOCOL**

**Study title:**

Virtual Reality vs. tablet video as a Pre-procedural Education Platform of Venipuncture in Pediatric Patients in Terms of Procedural Pain and Discomfort: a Prospective Randomized Trial

**Principal Investigator:**

Jin-Woo Park, MD, PhD

Department of Anesthesiology and Pain Medicine,

Medical Virtual Reality Research Group,

Seoul National University Bundang Hospital,

(e-mail: [jinul8282@gmail.com](mailto:jinul8282@gmail.com))

**Co-investigators:**

Jiyoun Lee, MD; Jung-Hee Ryu, MD, PhD; Sunghee Han, MD, PhD; Soo Hyun Seo, MD, PhD

1. **Summary**

Venipuncture is one of the most frequent and frightening medical procedures for children. Behavioral interventions to alleviate venipuncture pain are based on the gate-control theory which suggests attention, thoughts, and beliefs influence pain sensation. Distraction and education are widely adopted behavioral approaches. Recently, virtual reality (VR) systems have been utilized to reduce procedural pain or anxiety. However, to manage needle-related pain and anxiety, previous studies have used VR just as a distraction tool. This prospective, randomized, and clinical trial is designed to evaluate whether immersive pre-procedural VR education about venipuncture could reduce procedural pain and anxiety in children. Ninetty children scheduled for venipuncture procedure at the phlebotomy unit will be randomly divided into either the video group (n = 30) or VR group (n = 30). Children in the video group enter the phlebotomy unit with conventional simple instruction for venipuncture procedure, whereas those in the VR group receive a 4-min immersive VR education regarding the process of venipuncture. Pain and anxiety of pediatric patients will be evaluated with the Children’s Hospital of Eastern Ontario pain scale (CHEOPS). Parental satisfaction score, procedure time, incidence of repeated procedure, and process difficulty score rated by phlebotomists will be also recorded.

1. **Purpose**

To evaluate the effect of VR education of venipuncture process on pain and anxiety of pediatric patients and on efficiency of the procedure.

Hypothesis #1: VR education about venipuncture process before the procedure may reduce pain and anxiety in pediatric patients.

Hypothesis #2-a: VR education may improve parental satisfaction about the procedure.

Hypothesis #2-b: VR education may reduce procedure time

Hypothesis #2-c: VR education may reduce the requirement of needle re-insertion due to bad patient cooperation

Hypothesis #2-d: VR education may decrease the level of difficulty in performing venipuncture.

Hypothesis #2-e: VR education may increase parental satisfaction score.

1. **Background**

Venipuncture is one of the most frequently performed and also one of the most frightening procedures for pediatric patients. Unmanaged pain and anxiety associated with medical procedures might cause short-term suffering as well as negative long-term complications such as posttraumatic stress syndrome or needle phobia. Behavioral interventions to decrease venipuncture pain are based on the gate-control theory, which suggests attention, thoughts, and beliefs influence pain sensation. Distraction and education are widely adopted behavioral approaches.

Recently, VR systems have been introduced in patient education to reduce procedural pain or anxiety, and to improve outcome with the advance of technology. The VR with 360° video can deliver information via a consistent, vivid, and immersive experience to pediatric patients without physical and financial limitations. High immersion and vividity are the main characteristics provided by VR technology. Distraction using a VR game was reported to effectively reduce “Worst pain” and “Pain unpleasantness” during venipuncture in pediatric patients. VR systems providing procedural information through a simulated experience have been proven as an effective education platform to minimize peri-procedural anxiety in children. However, these previous studies have used VR just as a distraction tool to manage needle-related pain and anxiety.

1. **Inclusion Criteria, Exclusion Criteria**

**Inclusion:**

Aged 4–8 years, undergoing venipuncture at the phlebotomy unit in Seoul National University Bundang Hospital (SNUBH)

**Exclusion:**

History of prematurity or congenital disease

Hearing or vision impairment

Cognitive deficits or cognitive and intellectual developmental disabilities

History of epilepsy or seizure taking psychoactive medications

Prior experience of venipuncture for the past 1 year

1. **Targeted Number of Subjects and Calculation Basis**

In a pilot study of 40 pediatric patients (20 pairs) undergoing venipuncture, the CHEOPS score (mean [standard deviation]) for the video group was 7.7 (2.0) and 6.5 (1.7) for the VR group, respectively. A power analysis was carried out using G*Power 3.1.2 (Heinrich-Heine University, Düsseldorf, Germany). Based on the pilot data, a sample size of 45 patients per group was calculated to be necessary with a power of 0.8, a significance level of 0.05, and a 10% assumed dropout rate.

1. **Recruitment of research subjects**

Only researchers participating in this research can explain about the study and acquire informed consent from pediatric patients and their caregivers who will visit the phlebotomy unit of SNUBH to perform venipuncture procedure. Written informed consent will be obtained from all parents/guardians of pediatric patients, and children aged 7 years or older sign additional agreements directly after receiving detailed instructions with their parents/guardians. He/she can refuse to participate in the study at any time during the experience of VR education and the venipuncture process after obtaining the consent. The researcher will explain using a general term that can be fully understood by the subject who is not a medical person and will give enough information about the contents of this research, and the benefits and disadvantages from the research.

1. **Randomization**

The children are randomized to the video or VR group using a computer-generated randomization code (Random Allocation Software version 1.0; University of Medical Sciences, Isfahan, Iran), 10 min before venipuncture procedure. An opaque envelope containing sequential numbers is transferred to another researcher, and the intervention is performed in the separated area 5 min prior to entering the phlebotomy unit.

1. **Intervention**

For pediatric patients in the VR group receive a 4 min VR education about the procedure with a head- mounted VR display. Participants in the video group received a 4-min video education using a tablet PC. The contents were identical to that of the VR group, e.g., the contents used in the VR group were transformed into a 2-dimensional video.

1. **Virtual reality experience of the venipuncture procedure**

The VR experience is provided as a 360° 3-dimensional virtual environment that introduces and explains the process of venipuncture. The 4-min video was produced in collaboration with a VR producing company (JSC GAMES, Seoul, Korea). Chatan/Mona and Ace, famous animation characters of an animated film ‘Hello Carbot’ (ChoiRock Contents Factory, Seoul, Korea), explains the process of venipuncture in detail, encouraging the child to cooperate appropriately. Permission to use these animation characters have been obtained (licensing agreement with ChoiRock Contents Factory). In the VR system, pediatric patients experience the process of venipuncture with Chatan/Mona and Ace. The child can learn how to posture in front of phlebotomy desk and to cooperate appropriately through the VR education. A head-mounted VR display, Oculus Go (Oculus VR, Menlo Park, CA, USA) will be used to play the VR video.

1. **Outcome measurement**

Children’s pain and anxiety during the venipuncture procedure is measured with the children’s hospital of eastern ontario pain scale (CHEOPS) by a blinded single evaluator to exclude any possible interrater bias. Parents/guardians’ satisfaction score about the overall process of venipuncture using a numerical rating scale (11 NRS; 0, extremely dissatisfied; 10, extremely satisfied) will be recorded.

The total time for venipuncture procedure time from sitting at phlebotomy desk to the successful needle insertion for blood sampling) and the requirement of needle re-insertion due to bad patient cooperation are recorded by the blinded single evaluator. After the procedure, the phlebotomist will score the level of difficulty of performing the procedure of each child using a NRS (11 NRS; 0, extremely dissatisfied; 10, extremely difficult).

1. **Statistical analysis**

SPSS version 21.0 (SPSS Inc., IBM, Chicago, IL, USA) is utilized for all statistical analyses.

Continuous variables are indicated as median (interquartile range) or mean (standard deviation) according to the normality of the data. Categorical variables are presented as numbers (%). The Mann–Whitney U test was utilized to compare continuous study outcomes between the VR and video groups. Categorical outcomes were compared between the study groups using Fisher’s exact or chi-square test, as appropriate. We performed a multiple linear regression analysis to determine independent factors associated with the CHEOPS pain score.

1. **The benefits and risks**

In this study, it is considered that there is no additional risk or side effect due to the experience of less than 4 minutes VR experience training conducted in this study. In case of VR education experience, it is expected that positive effects on patient cooperation, and pain and anxiety during the procedure.

1. **Study stop or drop**

After the consent form is obtained, if the subject does not cooperate well or refuses to participate in the experience of video or VR education, and if the subject and guardian want to stop participating in the study, the study will be stopped and the subject will be dropped out of the study.

1. **Patients’ Consent**

- Who will provide consent: Study subjects and caregivers
- To minimize the possibility of forcible or unjustified effects: Avoid unfair deception, unreasonable pressure or intimidation. Obtaining agreement only after confirming that the subject has an adequate understanding of the participation and the opportunity to take full account of the participation in the study.
- Language that can be understood by research subject or parents: Korean language without difficult Chinese characters or English.

1. **Payment for participation**

There is no financial benefit for the study subjects involved in clinical trials. However, small toys are provided to the pediatric patients participating in the study as a gift.
