# Supplementary material for: Virtual reality vs. Tablet video for venipuncture education in children: A randomized clinical trial
Source: PLoS One. 2024 Aug 27;19(8):e0307488. doi: 10.1371/journal.pone.0307488 (PMC11349209; doi:10.1371/journal.pone.0307488)
Supplement: Attachment — Submitted filename: response to reviewers.docx [file pone.0307488.s005.docx]

**Journal requirements:**

When submitting your revision, we need you to address these additional requirements.

**1.** Please ensure that your manuscript meets PLOS ONE's style requirements, including those for file naming. The PLOS ONE style templates can be found at

https://journals.plos.org/plosone/s/file?id=wjVg/PLOSOne_formatting_sample_main_body.pdf and

<https://journals.plos.org/plosone/s/file?id=ba62/PLOSOne_formatting_sample_title_authors_affiliations.pdf>

Answer)

We appreciated your kind review. We have reviewed the journal's format and made the necessary revisions.

**2.** Thank you for stating the following financial disclosure:

“This work was supported by the National Research Foundation of Korea (NRF) grant funded by the Korea government (MSIT). (No. 2022R1F1A1072210).”

Please state what role the funders took in the study. If the funders had no role, please state: "The funders had no role in study design, data collection and analysis, decision to publish, or preparation of the manuscript."

If this statement is not correct you must amend it as needed.

Please include this amended Role of Funder statement in your cover letter; we will change the online submission form on your behalf.

Answer)

The funders had no role in study design, data collection and analysis, decision to publish, or preparation of the manuscript. We have revised the cover letter as advised.

3. We note that you have a patent relating to material pertinent to this article. Please provide an amended statement of Competing Interests to declare this patent (with details including name and number), along with any other relevant declarations relating to employment, consultancy, patents, products in development or modified products etc. Please confirm that this does not alter your adherence to all PLOS ONE policies on sharing data and materials, as detailed online in our guide for authors http://journals.plos.org/plosone/s/competing-interests by including the following statement: "This does not alter our adherence to PLOS ONE policies on sharing data and materials.” If there are restrictions on sharing of data and/or materials, please state these. Please note that we cannot proceed with consideration of your article until this information has been declared.

This information should be included in your cover letter; we will change the online submission form on your behalf.

Answer)

Sunghee Han and Jin-Woo Park are the co-inventors of the patent, “Medical experience in hospitals provided with VR or AR system”; Korean patent registration number: 1021115310000. No application of this patent, including development of medical devices or business models, is in progress or planned. This does not alter our adherence to PLOS ONE policies on sharing data and materials. As you recommended, we have attached the description of the patent in the cover letter.

4. In the online submission form you indicate that your data is not available for proprietary reasons and have provided a contact point for accessing this data. Please note that your current contact point is a co-author on this manuscript. According to our Data Policy, the contact point must not be an author on the manuscript and must be an institutional contact, ideally not an individual. Please revise your data statement to a non-author institutional point of contact, such as a data access or ethics committee, and send this to us via return email. Please also include contact information for the third party organization, and please include the full citation of where the data can be found.

Answer)

According to our institution's IRB (Institutional Review Board) regulations, we cannot upload the data from our research to a public server because we did not provide prior notification regarding the data sharing during patient consent for research participation. Therefore, instead of the co-authors of this manuscript, the IRB will act as the contact point for determining appropriate requests for data sharing. We will include the contact information, snubhirb@gmail.com, with an IRB approval number : B-2211-791-301 during the revised article submission.

5. We note that Figure 2 in your submission contain copyrighted images. All PLOS content is published under the Creative Commons Attribution License (CC BY 4.0), which means that the manuscript, images, and Supporting Information files will be freely available online, and any third party is permitted to access, download, copy, distribute, and use these materials in any way, even commercially, with proper attribution. For more information, see our copyright guidelines: http://journals.plos.org/plosone/s/licenses-and-copyright.

We require you to either (1) present written permission from the copyright holder to publish these figures specifically under the CC BY 4.0 license, or (2) remove the figures from your submission:

1. You may seek permission from the original copyright holder of Figure 2 to publish the content specifically under the CC BY 4.0 license.

We recommend that you contact the original copyright holder with the Content Permission Form (http://journals.plos.org/plosone/s/file?id=7c09/content-permission-form.pdf) and the following text:

“I request permission for the open-access journal PLOS ONE to publish XXX under the Creative Commons Attribution License (CCAL) CC BY 4.0 (http://creativecommons.org/licenses/by/4.0/). Please be aware that this license allows unrestricted use and distribution, even commercially, by third parties. Please reply and provide explicit written permission to publish XXX under a CC BY license and complete the attached form.”

Please upload the completed Content Permission Form or other proof of granted permissions as an "Other" file with your submission.

In the figure caption of the copyrighted figure, please include the following text: “Reprinted from [ref] under a CC BY license, with permission from [name of publisher], original copyright [original copyright year].”

2. If you are unable to obtain permission from the original copyright holder to publish these figures under the CC BY 4.0 license or if the copyright holder’s requirements are incompatible with the CC BY 4.0 license, please either i) remove the figure or ii) supply a replacement figure that complies with the CC BY 4.0 license. Please check copyright information on all replacement figures and update the figure caption with source information. If applicable, please specify in the figure caption text when a figure is similar but not identical to the original image and is therefore for illustrative purposes only.

Answer)

Thank you for the comment.

\# Fig 2A and Fig 2C

The cartoon characters appearing in Figure 2A and 2C of our paper were permitted for free use in accordance with CC BY 4.0. We cannot present PLOS ONE with the "written permission" from the copyright holder since the company that constructs our contents merged with another company, and the new company has no obligation to provide us with written permission.

However, the permissions for free use of both Figures in accordance with CC BY 4.0 were clearly documented in our previous articles: Front Med (Lausanne). 2022 Apr 7;9:849541 (for Fig 2C); J Clin Med. 2018 Sep 17;7(9):284; and JAMA Pediatr. 2019 Nov 1;173(11):1026-1031 (for Fig 2A).

So, it is evident that the two figures are free to use in accordance with CC BY 4.0.

However, it is true that we cannot submit written permission from the copyright holder to PLOS ONE for this article. So, the Chief Editor has the full right to request us to remove the figures from this article. If you do not allow us to include the figures in this article, we will rightfully remove them.

If you allow us to present the figures, we will add the description of the licensing agreement was added to the methods section and Fig 2 caption as follows ( as you can in this revision):

“We secured permission to use the cartoon characters through a licensing agreement with ChoiRock Contents Factory.” (page: 6)

“Reprinted from [“Hello Carbot”] under a CC BY license, with permission from [ChoiRock Contents Factory], original copyright [2017].” (page: 7)

\# Fig 2B (research participant’s photograph)

Regarding the use of the research participant’s photograph in Figure 2B, it has been approved by the parents of the child. A consent form for this will be attached as a separate file.

**Reviewer 1**

1) Firstly, could you elaborate more on the study protocol in the Methods section? While it is mentioned that a single-blinded researcher measured the CHEOP score and outcomes, I am curious about the actual study procedures. For instance, did children experience virtual reality or watch conventional videos in separate room and move to phlebotomy unit afterward? Additionally, what was the time interval between education and venipuncture? Also, one minor thing about your supplement data ‘protocol.docx’. While the total number of children is correctly indicated as 90, there seems to be an inconsistency in the allocation to both groups, where each is designated as 30.

Answer)

Thank you for your kind feedback. We performed the interventions in an independent room separated from the phlebotomy unit, and there was an interval of 5 min or less between education and venipuncture.

We have provided additional explanations in the Methods section as follows.

“Another independent researcher received a sealed envelope with the randomization number and performed the intervention in an independent room separated from the phlebotomy unit.” (page: 6)

“Immediately after the VR or video session, the patients were moved to the phlebotomy unit for venipuncture. The interval from the end of education to the positioning at the phlebotomy desk did not exceed 5 min.” (page: 7)

We have also added the description to “protocol.docx”.

2) Also, to establish the similarity between both groups, I recommend including p-values in Table 1 to support the claim about their comparable characteristics.

Answer)

Thank you for your suggestion. P-values have been included as advised.

3) I observed that the satisfaction scores from guardians were uniformly high, with most participants seems like giving a score of 10/10 in both groups. Given the limited variability in satisfaction scores, traditional statistical analyses may not be well-suited for such uniform data. I am interested in understanding the statistical approach used to assess and compare these high satisfaction scores. Moreover, considering the near-universal high ratings, it raises a broader question about the meaningfulness of comparing satisfaction levels between the two groups.

Answer)

Thank you for the advice. In fact, we drew attention to the fact that the results were contrary to those of a previous study that showed a significant difference in parental satisfaction between the VR group and the control group which received simple verbal education. Compared using different approaches, there was no significant difference in the parental satisfaction between the two groups. Active multimedia education to reduce procedural pain in pediatric patients might increase the satisfaction of the parents, in both groups. We added the description in the discussion section as follows.

“These results are contrary to those of a previous study that showed a significant difference in parental satisfaction between the VR group and the control group which received simple verbal education [15]. Active multimedia education to reduce procedural pain in pediatric patients might increase the satisfaction of the parents, in both groups.” (page: 14, line: 5-9)

4) Regarding the excluded patients, the figure 1 flowchart displays only those who did not agree to consent, while you mentioned other exclusion criteria. To adhere to the CONSORT guidelines, could you refine the number of excluded children in the flowchart accordingly?

Answer)

Thank you for your kind clarification. Upon review, I have found that the figure missing the excluded patients has been incorrectly registered. I will attach the newly revised Figure 1 flow chart. The "Patient" section in the Methods has also been updated accordingly. Thank you.

5) Moreover, I am interested in understanding the reasoning behind excluding children with a history of venipuncture in the previous year. Could you provide further details on this aspect?

Answer)

We appreciated the comment. When we planned the research, we hypothesized that if children had prior experience with the same procedure within the last year, it could potentially influence their perception of pain and anxiety associated with the procedure, either positively or negatively. To minimize any bias, children with the history were excluded from the study, as we did in our previous studies regarding VR experiential education.

6) Concerning the CHEOP score, I would appreciate it if you could clarify your rationale for dividing the score into three categories (mild/moderate/severe) and provide the reference for this categorization. Additionally, why did you choose the CHEOP score over other pain scale systems for children? Given that the CHEOP score was initially designed for children aged 1 to 5 years old and those who are postoperative, could you provide more explanation and references on why you specifically chose this scoring system?

Answer)

Thank you for the comment. In our current study, it was revealed that there is a statistically significant difference in CHEOP scores between the two groups. While this statistical difference is important, we also divided the data into thirds to visually demonstrate the distribution of pain among the children. There is no specific rationale for this division.

The CHEOPS (1) was developed for use in the postoperative setting for children one to 12 years of age, but has been applied to other acute pain situations, such as immunizations (2,3), venipuncture (4,5), fracture reduction ([6](https://www.ncbi.nlm.nih.gov/pmc/articles/PMC2706564/#b37-prm14047)) and laceration repair ([7](https://www.ncbi.nlm.nih.gov/pmc/articles/PMC2706564/#b38-prm14047)). CHEOPS has included the age range of our study, and is one of the most used behavioral assessment tools to evaluate pain caused by venipuncture in previous research. Accordingly, we decided to use CHEOPS in this study, as we did in our previous study (8).

1. McGrath PJ, Johnson G, Goodman JT, Dunn J, Chapman J. CHEOPS: A behavioral scale for rating postoperative pain in children. In: Fields HL, Dubner R, Cervero F, editors. *Advances in Pain Research and Therapy.* New York: Raven Press; 1985. pp. 395–402.

2. Cassidy KL, Reid GJ, McGrath PJ, et al. Watch needle, watch TV: Audiovisual distraction in preschool immunization. *Pain Med.* 2002;3:108–18.

3. Cassidy KL, Reid GJ, McGrath PJ, Smith DJ, Brown TL, Finley GA. A randomized double-blind, placebo-controlled trial of the EMLA patch for the reduction of pain associated with intramuscular injections in four to six-year-old children. *Acta Paediatr.* 2001;90:1329–36.

4. Galinkin JL, Rose JB, Harris K, Watcha MF. Lidocaine iontophoresis versus eutectic mixture of local anesthetics (EMLA) for IV placement in children. *Anesth Analg.* 2002;94:1484–8.

5. Van-Cleve L, Jonson L, Pothier P. Pain responses of hospitalized infants and children to venipuncture and intravenous cannulation. *J Pediatr Nurs.* 1996;11:161–7.

6. McCarty EC, Mencio GA, Walker LA, Green NE. Ketamine sedation for the reduction of children’s fractures in the emergency department. *J Bone Joint Surg Am.* 2000;82:912–8.

7. Klein EJ, Diekema DS, Paris CA, Quan L, Cohen M, Seidel KD. A randomized, clinical trial of oral midazolam plus placebo versus oral midazolam plus oral transmucosal fentanyl for sedation during laceration repair. *Pediatrics.* 2002;109:894–7.

8. Ryu JH, Han SH, Hwang SM, Lee J, Do SH, Kim JH, et al. Effects of Virtual Reality Education on Procedural Pain and Anxiety During Venipuncture in Children: A Randomized Clinical Trial. Front Med (Lausanne) 2022;9:849541.

**Reviewer 2**

1) What test was used for the sample size calculation? Is that a two-sample t-test? Why not use Mann-Whitney as the primary analysis does?

Answer)

Thank you for the comment. We used a two-sample t-test to calculate the sample size. This was because that the number of samples in our study was 45 people per group, so we expected CHEOPS score to show normal distribution. However, the variable did not satisfy normal distribution, so the Mann-Whitney test was used for statistical analysis instead. Of course, even if Mann-Whitney was used to calculate the sample size, there would not have been a sufficient difference in the total number of participants, and the statistical results would not have been different from the current results.

2) Can you calculate a Spearman correlation between VAS and the pain scale, overall and by education group? Is there a significant interaction between VAS and group in the regression?

Answer)

As you recommended, we performed Spearman correlation analysis between baseline pain expectation (VAS) and the CHEOPS. We found a positive and significant correlation; the Spearman correlation coefficient was 0.477 (*P* < 0.001). We also performed Spearman correlation analysis between overall and by education group. Except for CHEOPS, no variables showed significant correlation with education group. The Spearman correlation coefficient between the education group and pain expectation (VAS) was -0.012 (*P* = 0.912).
